# Application of enhanced recovery after surgery following liver transplantation

**DOI:** 10.1186/s12957-023-03139-x

**Published:** 2023-08-17

**Authors:** Boxun Jin, Yanmei Gu, Shuangmei Xi, Xin Liu, Xiulian Wu, Guangming Li

**Affiliations:** Department of Intensive Care Unit, Beijing Youan Hospital, Capital Medical University, No. 8 Yoanmenwai Xitoutiao, Beijing, 100069 China

**Keywords:** Liver transplantation, Enhanced recovery after surgery, Liver cancer, Liver failure, Liver cirrhosis

## Abstract

**Objective:**

To investigate the effect of an enhanced recovery after surgery (ERAS) programme following liver transplantation and to further clarify the safety and clinical application value of an ERAS programme.

**Methods:**

A retrospective analysis of 250 patients who underwent liver transplant at Beijing You’an Hospital affiliated to Capital Medical University between March 2019 and December 2021 was conducted. According to different perioperative management methods, patients were divided into a control group (120 cases) and an ERAS group (130 cases). Postoperative safety indicators, efficacy indicators and economic indicators were compared between the two groups.

**Results:**

There was no significant difference in the safety indicators between the two groups. The ERAS group showed significantly lower results compared with the control group in terms of ventilator-associated pneumonia, urinary tract infection, pressure injury of oral and nasal mucosa, postoperative pain score 5 days after surgery and the incidence of delirium, whereas the Barthel score 10 days after surgery was significantly higher. There was no significant difference between the two groups in skin pressure injury or the Subjective Global Assessment grade 10 days after surgery. The length of intensive care unit stay, the total length of stay after surgery and the 10-day medical expenses after surgery were significantly lower in the ERAS group than in the control group.

**Conclusion:**

The application of an ERAS programme after liver transplantation can effectively promote the postoperative recovery of patients and reduce medical costs. Studies have shown that the ERAS programme has important application value in improving the postoperative quality of life and reducing the economic burden of patients after liver transplantation. This programme provides a new concept for related clinical improvement and application.

## Introduction

In 2001, the enhanced recovery after surgery (ERAS) research association was established in Europe and formally proposed the concept of ERAS [1], which is based on evidence-based medicine. Through the multidisciplinary collaboration of surgery, anaesthesia, nursing and nutrition, the clinical pathway of perioperative treatment is optimised to reduce perioperative stress responses and postoperative complications, shorten postoperative hospital stays and promote the rapid recovery of patients [2, 3]. Since the concept of ERAS was introduced into China in 2000, it has been applied in orthopaedics, breast surgery, gynaecology and gastrointestinal surgery and has achieved remarkable results [4–7].

Liver transplantation is an effective treatment for advanced liver diseases such as liver cancer, liver failure and cirrhosis. Compared with other surgical operations, liver transplantation is a difficult operation, involving a long surgery time, a high degree of trauma and a high incidence of postoperative complications. Coupled with the use of immunosuppressants, the perioperative management of liver transplantation is complicated. It is crucial to optimise the perioperative management strategy for patients undergoing liver transplant, accelerate the recovery of patients and improve the postoperative survival rate. There is currently no accepted standard protocol for ERAS after liver transplantation. Many previous studies on ERAS after liver transplantation have focused on a specific problem in the recovery process of patients, such as the early removal of tracheal intubation, early enteral nutrition, analgesia and sedation, or fluid management, but they have not adopted a comprehensive approach. Some domestic transplant centres have begun to explore the bundled ERAS scheme after liver transplantation and have achieved positive results, but the evaluation system is not perfect, and indicators, such as mortality, organ failure, hospital stay or cost, have been too macroscopic [8–12].

In response to the above two problems, the team has constructed a new bundled ERAS treatment and management programme after liver transplantation using standardised evidence-based medicine and evidence-based nursing methods, involving respiratory therapy, nutritional therapy, and mental and psychological treatment; by also including treatments from other disciplines, such as anaesthetic pain treatment and critical care, a comprehensive ERAS programme for patients has been created. This study retrospectively analysed the clinical data of 250 recipients of liver transplant who were admitted to Beijing You’an Hospital affiliated with Beijing University of Science and Technology between March 2019 and December 2021 and explored the effectiveness and safety of an ERAS bundled management programme after liver transplantation.

## Materials and methods

### Participants

This is a retrospective cohort study. The clinical data of 250 patients who underwent liver transplantation in our hospital between March 2019 and December 2021 were retrospectively analysed. All 250 patients received deceased donor liver transplantation. According to different perioperative treatments, they were randomly divided into a control group (120 cases) and an ERAS group (130 cases). Using the methods of evidence-based medicine and evidence-based nursing, our team constructed a bundled ERAS treatment and nursing programme after liver transplantation and applied it to the ERAS group. The control group received conventional perioperative management protocols. The detailed protocol is provided in Table 1. This study has been approved by the Ethics Committee of Beijing You’an Hospital affiliated with Capital Medical University (ethics number: LL-2022–020-K), and all patients and their families gave signed informed consent.

**Table 1:** The diagnosis and treatment measures of the ERAS group and the control group

	ERAS group	Control group
Psychological care	The application of multi-mode, individualised plan and targeted health education after operation. In order to reduce unnecessary contact during the epidemic, after the patient regains consciousness after the operation, the patient and relatives can be met regularly through the mobile terminal video connection	Routine postoperative health education. Face-to-face bedside visits are prohibited due to lockdown management requirements during the pandemic. Unusual video meeting
Pipeline management		
Endotracheal intubation and follow-up respiratory support	Comprehensive assessment of the condition, in the absence of serious postoperative complications and clear indications for mechanical ventilation, the SBT process of disconnection from invasive mechanical ventilation and removal of orotracheal intubation was completed within 24 h to achieve off-line extubation. Immediately after weaning and extubation, transnasal high-flow oxygen therapy was administered	Comprehensive assessment of the condition, in the case of patients with no deterioration of liver function and clear infection after surgery, the SBT process is completed. Ordinary nasal cannula or nebulised mask oxygen therapy was given after weaning and extubation
Gastric tube	Fully assess the patient’s level of consciousness, nutritional needs, gastrointestinal integrity and function. If the patient’s consciousness recovered well and there was no absolute indication for gastrointestinal decompression, the nasogastric tube was removed on the second postoperative day. If the evaluation found that there was a need for continued retention of the gastric tube, the tube was not removed	Assess the patient’s level of consciousness, nutritional needs, gastrointestinal integrity and function and remove the nasogastric tube after the patient’s consciousness is restored, the anus is exhausted, and there are no other absolute indications for retaining gastrointestinal decompression
Urinary catheter	The urinary catheter was removed 24 h after surgery. In the early stage, the urine was collected in a fresh-keeping bag, and in the later stage, the patient collected urine in a urinal by himself	The patient can remove the catheter after getting out of bed
Nutritional support		
When and how	Early nutritional support was initiated within 24 h after surgery when hemodynamically stable. Enteral nutrition or other food is given first through a nasogastric tube (when the tube is not removed) or by mouth. If there are counter-indications for enteral nutrition (abdominal compartment syndrome, severe diarrhoea, hemodynamic instability, or other dramatic changes), reduce the dose of nutritional enteral nutrition or discontinue enteral nutrition. Please consult the nutrition department, and according to the nutritional needs of patients, appropriate intravenous nutritional supplements are given to avoid nutritional deficiencies or excessive nutrition	Complete parenteral nutrition was given in the early stage, and after the gastrointestinal function recovered, partial enteral nutrition was started, and intravenous nutrition support was gradually withdrawn. If there are counter-indications for enteral nutrition (abdominal compartment syndrome, severe diarrhoea, hemodynamic instability, or other dramatic changes), reduce the dose of nutritional enteral nutrition or discontinue enteral nutrition
Nasogastric tube feeding parameters	The initial feeding speed is 20 ml/h, and the dosage is gradually increased; the temperature of the nutrient solution is 40 °C; the target feeding amount is 30 kcal/kg/day	
Oral feeding process	Liquid food–semi-liquid food–normal diet	
Early activity	After returning to the ICU after the operation, if there is no special need, the upper body is immediately raised by 30°. When the patient regains consciousness but has not yet been released from the ventilator, assist the patient to turn over on the bed and give training such as fisting, arm raising, ankle pumping and lower limb pedalling. After the patient is released from the ventilator, assist the patient to gradually increase the range of exercise, including standing at the bedside, getting out of bed and sitting, and walking with assistance	Regular activities in the ICU, including turning over once every 2 h, physical therapy on the chest, maintaining the functional position of both lower extremities 2 times a day, limb massage, activities, etc
Pain relief		
Evaluation method	The visual analogue scale and numerical rating scale were used to evaluate the pain intensity of patients at rest and during exercise, and the treatment effect was also evaluated	The visual analogue scale and numerical rating scale were used to evaluate the pain intensity of patients at rest and during exercise, and the treatment effect was also evaluated
Analgesic mode	Intravenous analgesia pump, used immediately after surgery, for a duration of 48 h. Other strong opioid analgesics can be given if the tracheal tube is not removed and dehumidified	Analgesic pump, used immediately after surgery for a duration of 48 h. Other strong opioid analgesics can be given if the tracheal tube is not removed and dehumidified
	Administer weak opioids + NSAIDs 2–5 days after the operation with analgesia pump	
	Sequential analgesia with NSAIDs	

*ERAS* enhanced recovery after surgery, *ICU* intensive care unit, *NSAIDs* nonsteroidal anti-inflammatory drugs

### Inclusion and exclusion criteria

The inclusion criteria for recipients were the following: (1) complete clinical data, preoperative diagnosis with clear indications for liver transplantation and approval by the hospital transplant ethics committee; (2) elective allogeneic orthotopic liver transplantation; and (3) voluntary participation in the study and signed informed consent. The exclusion criteria were as follows: (1) patients undergoing emergency liver transplantation, (2) patients undergoing re-liver transplantation, (3) patients undergoing combined liver and kidney transplantation or liver transplantation combined with other operations, (4) patients who died during the operation, (5) patients who were automatically discharged or moved to another hospital, and (6) critically ill patients with postoperative primary graft failure or delayed graft recovery, postoperative severe bleeding, acute renal failure, acute respiratory distress syndrome or acute dysfunction of other organs.

### Observation indicators

General information: sex, age, body mass index (BMI), diagnosis, model of end-stage liver disease (MELD) score [13], cirrhosis Child score [14], preoperative subjective comprehensive nutritional assessment scale (Subjective Global Assessment [SGA] classification) [15], operation time, intraoperative blood loss and anhepatic period of liver transplantation. In addition, the composition of the preoperative diagnosis of the patients was observed.

Safety indicators: The incidence of postoperative complications (accidental extubation, re-tracheal intubation, ward falls, acute urinary retention and airway aspiration).

Efficacy indicators: Incidence of ventilator-associated pneumonia, incidence of urinary tract infection, incidence of skin pressure injury, incidence of pressure injury of oral and nasal mucosa, SGA grading 10 days after surgery, activities of daily living scale score 10 days after surgery (Barthel index) [16], postoperative pain score 5 days after surgery (visual analogue scale) [17] and incidence of delirium.

Economic indicators: Intensive care unit (ICU) length of stay, total postoperative hospital stay, hospitalisation costs and readmissions within 90 days.

### Statistical analysis

Statistical analysis was performed using SPSS 19.0 software (IBM Corporation, Armonk, NY, USA). Measurement data were expressed as mean ± standard deviation ($\overset{-}{x}\pm{s}$ ) or median and full range. According to the results of the normal distribution test and homogeneity of variance test, either the *t*-test or rank combined analysis was used. Enumeration data were expressed as cases (%), and the *χ*^2^ test was used for comparison between groups. A score of *p* < 0.05 was considered statistically significant.

## Results

### General information

There were 120 cases in the control group, with a male to female ratio of 17:13 and an average age of 45 years (range 23–67 years); there were 130 patients in the ERAS group, with a male to female ratio of 41:24 and an average age of 49 years (range 22–63 years). There was no significant difference between the two groups in sex ratio, age, BMI, MELD index, Child score, preoperative SGA grade, operation time, intraoperative blood loss or anhepatic phase (data shown in Table 2). In the ERAS group, there were 73 patients with decompensated cirrhosis, 35 patients with primary liver cancer in the liver function compensation stage and 22 patients with various types of liver failure; in the control group, the numbers with these conditions were 71 patients, 31 patients and 18 patients, respectively. There was no significant difference between the preoperative diagnoses of the two groups (data shown in Table 3).

**Table 2:** Comparison of general data, preoperative condition and surgical conditions between ERAS group and control group

	Sex ratio (male/female)	Age (years)	BMI (kg/m^2^)	MELD index	Child score	Preoperative SGA grading cases (A/B/C)	Operation time (h)	Intraoperative blood loss (ml)	Anhepatic period (min)
ERAS Group (*n* = 130)	82/48	49, 22–63	23.87 ± 2.65	18.5 ± 4.1	10.5 ± 2.6	49/68/13	6.2 ± 1.9	850, 300–1700	45, 25–80
Control group (*n* = 120)	68/52	45, 23–67	22.53 ± 1.75	18.2 ± 7.1	10.8 ± 3.7	46/60/14	6.9 ± 1.5	1100, 300–1500	35, 30–70
*p* value	0.301	0.343	0.118	0.551	0.822	0.89	0.329	0.342	0.087

Data presented as median, full range or $\overset{-}{x}\pm{s}$ . *ERAS* enhanced recovery after surgery

*BMI* body mass index, *MELD* model for end-stage liver disease, *SGA* subjective global assessment

**Table 3:** Composition of preoperative diagnosis in the ERAS group and control group

	ERAS group (*n*)	Control group (*n*)
Decompensated cirrhosis	73	71
Primary liver cancer in liver function compensation stage	35	31
Various types of liver failure	22	18
Total	130	120

Decompensated cirrhosis is defined as an acute deterioration in liver function in a patient with cirrhosis and is characterised by jaundice, ascites, hepatic encephalopathy, hepatorenal syndrome or variceal haemorrhage [18]

*ERAS* enhanced recovery after surgery

### Safety indicators

As shown in Table 4, in the ERAS group, 6 patients had accidental extubation, 17 patients received re-tracheal intubation, 2 patients had acute urinary retention and there was 1 case with airway aspiration; in the control group, the numbers with these complications were 7 patients, 13 patients, 3 patients and 2 patients, respectively. There was no significant difference in safety indicators between the two groups, and no patient from either group fell in the ward.

**Table 4:** Comparison of safety data between the ERAS group and control group

	Accidental extubation (*n*)	Re-tracheal intubation (*n*)	Ward fall (*n*)	Acute urinary retention (*n*)	Airway aspiration (*n*)
ERAS group	6	17	0	2	1
Control group	7	13	0	3	2
*P* value	0.665	0.585	–	0.597	0.515

*ERAS* enhanced recovery after surgery

### Effectiveness indicators

In the ERAS group, there were 13 cases of ventilator-associated pneumonia, 4 cases of urinary tract infection and 1 case of oronasal mucosal pressure injury, which constituted a significantly lower number of cases than in the control group. There was no significant difference in skin pressure injury between the two groups. There was also no significant difference in SGA grading between the two groups 10 days after surgery, but the proportion of grade A in the ERAS group was 0.35, which was significantly higher than that of the control group (0.23). The Barthel score in the ERAS group 10 days after surgery was 71, which was significantly higher than that in the control group. The pain score in the ERAS group 5 days after surgery was 3.5, and delirium was observed in 10 patients, both of which were significantly lower than in the control group. Three patients in the ERAS group and two patients in the control group had low arterial flow within 2 weeks of surgery, but no definite findings of arterial thrombosis or anastomotic stenosis were found. All five patients received active anticoagulant therapy but did not receive thrombolysis, arterial catheter intervention or surgical treatment. None of the 250 patients had portal vein stenosis, portal embolus, bile duct stenosis or biliary fistula within 90 days of surgery.

The above results are shown in Table 5.

**Table 5:** Comparison of efficacy indicators between the ERAS group and control group

	Ventilator-associated pneumonia (*n*)	Urinary tract infection (*n*)	Skin pressure injury (*n*)	Oronasal mucosal pressure injury (*n*)	SGA classification 10 days after surgery (*n*)				Barthel index score 10 days after surgery	5-day postoperative pain score (*n*)	Delirium (*n*)
					A	B	C	Ratio of A grade			
ERAS group	13	4	3	1	45	68	17	0.35	71	3.5	10
Control group	28	13	8	9	28	69	23	0.23	54	6.4	19
*P* value	0.017	0.015	0.093	0.017	0.107			0.05	0.023	0.015	0.045

*ERAS* enhanced recovery after surgery, *SGA* subjective global assessment

### Economic indicators

Comparing the ICU length of stay, total postoperative hospital stay and 10-day postoperative medical expenses between the two groups, the results for the ERAS group were lower than those of the control group, and the differences were statistically significant. There was no significant difference between the ERAS group and the control group in terms of readmission at 90 days after surgery. The above results are shown in Table 6.

**Table 6:** Comparison of economic data between the ERAS group and control group

	ICU length of stay (days)	Total hospital stay after surgery (days)	90-day readmission and non-discharge (*n*)	Postoperative hospitalisation expenses (1000 $) (*n*)
ERAS group	2.4	13.4	17	7.0, 2.7–17.9
Control group	4.1	18.3	25	12.8, 5.0–33.9
*P* value	0.042	0.013	0.101	0.01

*ERAS* enhanced recovery after surgery, *ICU* intensive care unit

### Analysis of survival rate

**Fig. 1:**
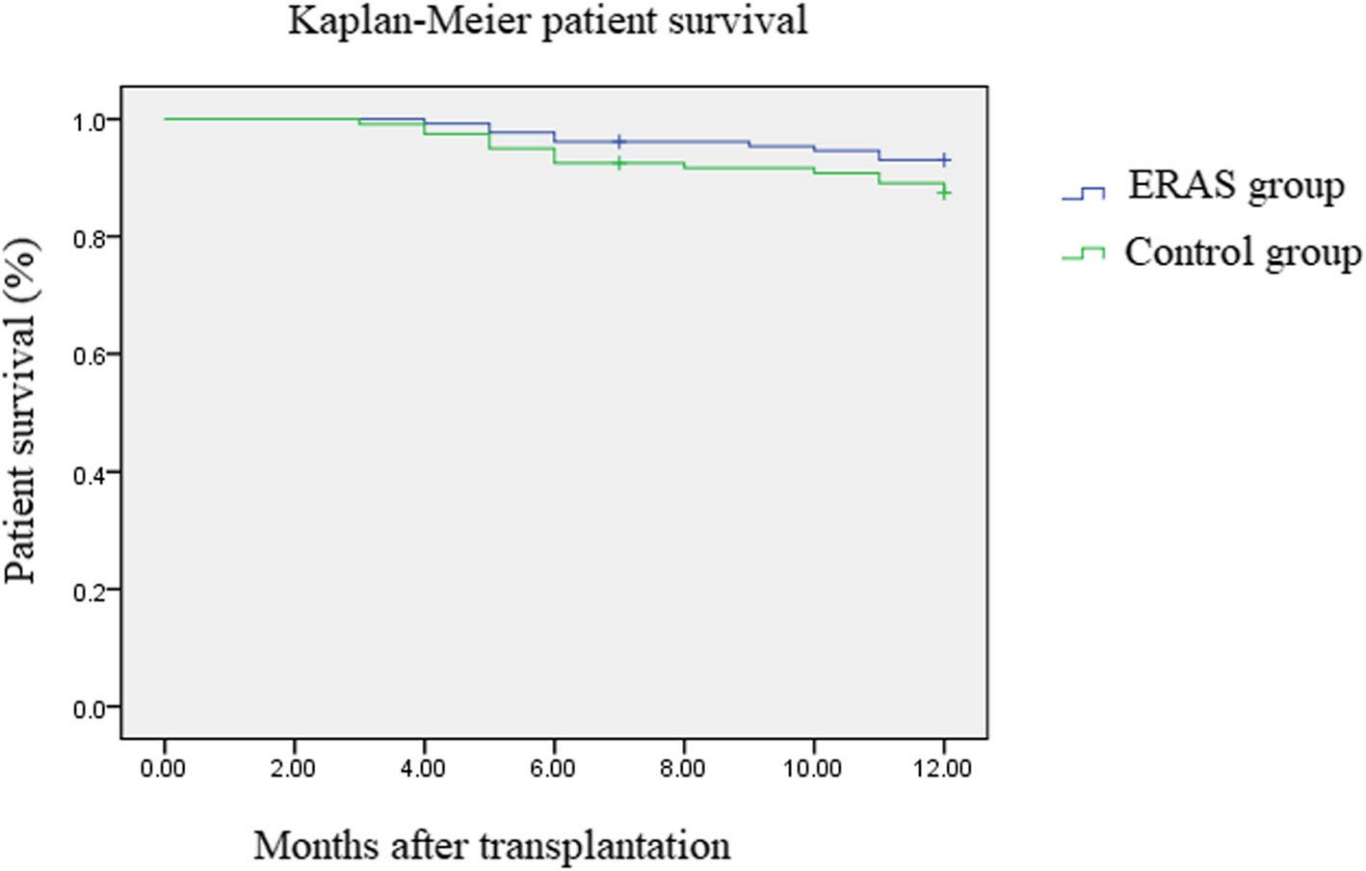
Kaplan–Meier analysis of overall survival in two groups of patients

In all 250 patients who underwent liver transplantation, the operation was completed successfully (completion rate 100%). The 250 patients were followed up for 12 months; of the 130 patients in the ERAS group, 9 patients died, whereas out of the 120 patients in the control group, 15 patients died. The survival analysis curve is shown in Fig. 1. The survival curves of the two groups were compared as a whole, with no statistical significance (*p* = 0.131).

## Discussion

In this study, using standardised evidence-based medicine and evidence-based nursing methods, a new bundled ERAS treatment and management programme after liver transplantation were constructed, involving respiratory therapy, nutritional therapy, psychotherapy, anaesthetic pain treatment, critical care and other disciplines. This study revealed that there were no significant differences in the safety indicators between the two groups, but the ERAS group exhibited significantly better results than the control group in terms of ventilator-associated pneumonia, urinary tract infection, pressure injury of the oral and nasal mucosa and malnutrition. The length of stay in the ICU, the total length of hospital stay after surgery and the 10-day medical expenses after surgery were lower in the ERAS group, which effectively reduced medical costs.

Previous studies have shown that an ERAS programme can be effective, and this is closely related to the patient’s baseline state. A study published by Wang et al. in 2018 showed that during the application of an ERAS programme after hepatectomy, poor liver function and intraoperative bleeding were the risk factors most likely to result in failure of the postoperative ERAS programme [19]. Hepatectomy has a smaller impact on patients than liver transplantation, and the overall condition of patients before hepatectomy is better than that of patients undergoing liver transplantation; the postoperative recovery is more stable and rapid, and the treatment is relatively simpler. However, the ERAS programme for hepatectomy cannot be directly applied to liver transplant. A large number of studies have demonstrated that liver transplantation is a treatment method with a high complication rate and many uncertain prognostic factors [20], and patients may have more risk factors leading to failure in the application of the ERAS programme. At present, the research on bundled ERAS treatment after liver transplantation is still in its infancy, and there are no established ERAS measures for patients with special conditions. Therefore, we excluded patients who were critically ill and tried to ensure that the patients in the two groups were similar in terms of baseline condition and operation process and were balanced in other aspects to avoid the influence of preoperative and intraoperative confounding factors on postoperative treatment effect.

Safety during diagnosis and treatment is the primary issue for the implementation of ERAS programmes; however, many studies have placed equal importance on the safety and efficacy of ERAS treatments [21–23]. In this study, combined with the risks associated with multidisciplinary treatment measures after transplantation, the safety indicators of accidental extubation, re-tracheal intubation, ward falls, acute urinary retention and airway aspiration were used. These indicators are relatively serious acute complications after liver transplantation and are related to excessive extubation, postoperative exercise and oral feeding. By comparing the differences in the above incidence rates between the two groups, it was found that there was no significant difference in the safety indicators between the two groups; therefore, the bundled ERAS measures after liver transplantation developed in this study are reliable and safe.

Prolonged mechanical ventilation, prolonged immobilisation in bed, prolonged parenteral nutrition and prolonged retention of gastric and urinary catheters after liver transplantation can lead to a series of complications [24], including ventilator-associated pneumonia, urinary tract infection, pressure injury of the oral and nasal mucosa and malnutrition. In addition, various invasive procedures, restraint therapy and the above-mentioned related complications after liver transplantation can cause severe pain and even induce delirium [25], threatening the safety and life of patients. The bundled ERAS treatment plan shortens the time of invasive treatment to within a reasonable range, provides analgesic treatment and encourages patients to exercise for rehabilitation. Comparing the efficacy indicators of the two groups of patients, it can be seen that the ERAS group had a lower incidence of complications, better nutritional status, better pain control and better overall life outcomes. These results suggest that the ERAS programme can effectively solve the problems of excessive trauma, reduced body function, excessive pain and decreased activities of daily living caused by traditional treatment. In addition, it should be noted that there was no significant difference in the incidence of skin pressure injury between the two groups of patients, which may be due to the critical condition of the patients after surgery. The occurrence of skin pressure injury is not only related to local long-term compression but also to the patient’s soft tissue oedema; patients undergoing liver transplantation are generally in critical condition before surgery and often have long-term malnutrition, hypoproteinaemia and volume overload after surgery. In other cases, tissue oedema is obvious, and the skin is prone to pressure injury. Even when an ERAS programme is adopted, the preventive effect on skin pressure injury may not be satisfactory [26]. This also shows that although the ERAS programme can shorten the invasive treatment time, promote the recovery of patients and reduce the incidence of recent complications, its efficacy is limited by the overall condition of the patient. In patients with schizophrenia, the ERAS regimen is also less effective.

The final part of this study compared the differences in ICU stay time, total postoperative hospital stay and 10-day postoperative hospitalisation cost between the two groups, and the results suggested that ERAS measures resulted in a significant economic advantage. The reason is that, in the ERAS group, the incidence of complications was greatly reduced by optimising the postoperative invasive treatment time of liver transplant patients, rationally applying analgesic drugs and actively giving patients rehabilitative treatment. This is an effective solution to improve rehabilitation efficiency and reduce medical expenditure, which is consistent with the research results of other transplant centres in China [9–11]. However, it should be noted that the long-term prognosis of patients is the result of a combination of factors [27–29], and the short-term postoperative treatment has little effect on the long-term prognosis after the operation. There was no significant difference in the 90-day readmission rate between the two groups in this study. Rodriguez-Laiz et al. followed up liver transplant patients for 6 years after receiving ERAS, and the results showed that the readmission rate of patients receiving ERAS treatment was significantly reduced within 30 days [30]. In this study, patients were not followed up for such a long time, but the 12-month follow-up has shown that the ERAS regimen has no significant effect on the long-term prognosis of patients. In addition to early postoperative ICU treatment, the long-term prognosis of patients following liver transplant is also related to other factors, including rejection, being immunocompromised, tumour and liver disease recurrence and metabolic diseases. The 90-day postoperative hospitalisation of patients may be more affected by these factors and less related to whether the ERAS programme is implemented.

Our results preliminarily confirm that the ERAS programme can improve the recovery of patients following liver transplant. However, since this study is a single-centre retrospective study, the sample size is limited and the patients involved are from one hospital; therefore, the generalisability of the research results needs to be further verified. To better evaluate generalisability, large-scale multi-centre prospective studies should be conducted to expand the sample size and case type. In this way, the efficacy of the ERAS programme in different liver transplantation centres and patients with different types of liver transplantation can be observed. In this study, the formulation of the ERAS programme itself was based on evidence-based nursing. The author speculates that the ERAS programme is also applicable in different transplant centres and to a wider population, but this needs to be assessed; if future multi-centre studies also confirm that the ERAS programme can improve patient outcomes, the generalisation of our single-centre study results will be supported. At present, this study can only be used as a preliminary investigation to provide a reference for subsequent research.

The post-liver transplantation bundled ERAS scheme proposed in this study was developed by team members through the systematic and standardised retrieval, evaluation, evidence grading, review and finalisation of evidence-based medicine and evidence-based nursing literature and is highly comprehensive and scientific. During the implementation of the research, tasks were divided among the team members, who cooperated and implemented the diagnosis and treatment nursing measures in strict accordance with the bundled ERAS plan; the results are therefore objective and accurate.

## Conclusion

In conclusion, the application of the ERAS programme can shorten the ICU and hospitalisation time of patients, reduce the incidence of postoperative complications and reduce the cost of postoperative hospitalisation. Our results demonstrate that the ERAS programme, which can also effectively reduce the postoperative burden of patients and improve survival outcomes, has a high clinical application value, and it provides reliable research evidence for the promotion of the ERAS programme in the field of liver transplantation. However, in view of the retrospective nature of this study and the limitation of the small sample size, prospective studies with larger samples should be conducted in the future to further clarify the impact of the ERAS programme on long-term follow-up after liver transplantation.

## Data Availability

All data generated or analysed during this study are included in this article. Further enquiries can be directed to the corresponding author.

## Abbreviations

ERAS: Enhanced recovery after surgery
SGA: Subjective global assessment
ICU: Intensive care unit
BMI: Body mass index
MELD: Model of end-stage liver disease
VAS: Visual analogue scale
